# The Impact of HLA Class I-Specific Killer Cell Immunoglobulin-Like Receptors on Antibody-Dependent Natural Killer Cell-Mediated Cytotoxicity and Organ Allograft Rejection

**DOI:** 10.3389/fimmu.2016.00585

**Published:** 2016-12-19

**Authors:** Raja Rajalingam

**Affiliations:** ^1^Immunogenetics and Transplantation Laboratory, Department of Surgery, University of California San Francisco, San Francisco, CA, USA

**Keywords:** antibody-mediated rejection, antibody-dependent cell-mediated cytotoxicity, human leukocyte antigen, killer cell immunoglobulin-like receptors, natural killer cells, donor-specific antibodies, solid organ transplantation, transplant rejection

## Abstract

Natural killer (NK) cells of the innate immune system are cytotoxic lymphocytes that play an important roles following transplantation of solid organs and hematopoietic stem cells. Recognition of self-human leukocyte antigen (HLA) class I molecules by inhibitory killer cell immunoglobulin-like receptors (KIRs) is involved in the calibration of NK cell effector capacities during the developmental stage, allowing the subsequent recognition and elimination of target cells with decreased expression of self-HLA class I (due to virus infection or tumor transformation) or HLA class I disparities (in the setting of allogeneic transplantation). NK cells expressing an inhibitory KIR-binding self-HLA can be activated when confronted with allografts lacking a ligand for the inhibitory receptor. Following the response of the adaptive immune system, NK cells can further destroy allograft endothelium by antibody-dependent cell-mediated cytotoxicity (ADCC), triggered through cross-linking of the CD16 Fc receptor by donor-specific antibodies bound to allograft. Upon recognizing allogeneic target cells, NK cells also secrete cytokines and chemokines that drive maturation of dendritic cells to promote cellular and humoral adaptive immune responses against the allograft. The cumulative activating and inhibitory signals generated by ligation of the receptors regulates mature NK cell killing of target cells and their production of cytokines and chemokines. This review summarizes the role of NK cells in allograft rejection and proposes mechanistic concepts that indicate a prominent role for KIR–HLA interactions in facilitating NK cells for Fc receptor-mediated ADCC effector function involved in antibody-mediated rejection of solid organ transplants.

## Antibody-Mediated Rejection of Organ Allograft

The major hurdle to successful organ transplantation is graft rejection, a process orchestrated by sophisticated cell and antibody-mediated defense mechanisms, which has evolved primarily to combat invading microbes or diseased and damaged cells. The T cell-targeted immunosuppressive regimens (including T cell-specific antibodies, calcineurin inhibitors, mycophenolic acid, rapamycin, and prednisone) have effectively reduced the incidence of cell-mediated transplant rejection and have substantially improved 1-year graft survival to 88% following renal transplantation (1). Nevertheless, alloantibodies mediate a substantial proportion of the remaining graft rejection episodes, contributing to both early and late graft loss, particularly in sensitized populations such as patients with previous transplants and patients who have previously had multiple pregnancies or multiple blood transfusions (2).

Antibody-mediated rejection (ABMR) is recognized to be a key problem in organ transplantation and a major cause of late graft loss (3). Based on time course, the ABMR is classified as hyperacute, acute, or chronic (1). Hyperacute rejection, the first rejection phenotype observed in human organ transplantation, occurs immediately on perfusion of the transplanted organ with the blood of the recipient (4). Preformed donor-specific antibodies (DSAs) in recipient’s blood bind to antigens expressed on vascular endothelium of the transplanted allograft [such as human leukocyte antigens (HLAs), ABO blood group antigens, and other endothelial antigens] and trigger a cascade of complement activation, which results in tissue injury involving blood vessel wall damage, hemorrhage, neutrophil infiltration, platelet, and fibrin deposition. Reliable cross-matching methods and screening recipients for preformed circulating HLA antibodies to the prospective donor have almost eliminated the incidence of this devastating phenotype (5, 6).

Acute ABMR occurs at any time from days to years following transplantation, and results from DSA that may be preexisting or develop *de novo* after transplantation (7). At present, acute ABMR is defined by four criteria: clinical evidence of acute graft dysfunction, histologic evidence of acute tissue injury, immunohistologic evidence for the action of DSAs (C4d deposition in peritubular capillaries), and DSAs detected in the serum (8). ABMR occurs in 6.7% of renal transplant patients and is present in approximately one-third of renal transplant patients diagnosed with acute rejection (9–11). Acute ABMR is characterized by a rapid rise in serum creatinine and is resistant to therapy with steroids or T cell-specific reagents.

Chronic ABMR develops over months or years before there are signs of graft dysfunction and is mediated by antibodies that develop *de novo*. The features of chronic ABMR in renal allografts include the following: duplication of the glomerular basement membrane, intimal cell proliferation of arterioles and infiltration with mononuclear cells, and lamination of the peritubular capillary basement membrane, which occurs together with the deposition of C4d in peritubular capillaries and glomeruli. Chronic ABMR is the result of cumulative damage to the kidney and over 50% of recipients develop chronic ABMR at 10 years after transplantation (12). A further category of rejection, subclinical rejection, has recently been recognized, and this refers to pathological injury in the graft that has been caused by antibody and/or T cells, but which has not yet resulted in graft dysfunction.

## Mechanisms Involved in Antibody-Mediated Rejection of Organ Allograft

**Figure 1:**
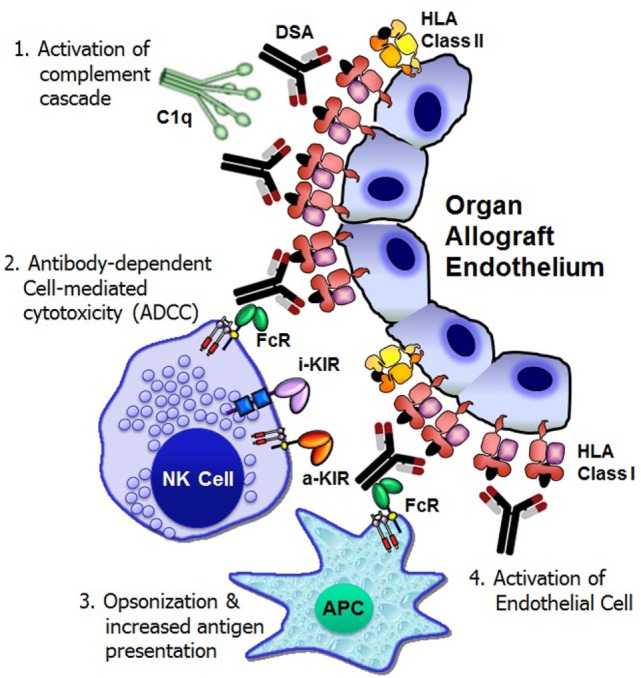
**Mechanisms of donor-specific antibody-mediated rejection of renal allografts**. Donor-specific HLA antibody binding to the allograft endothelium may trigger four distinct cellular and humoral mechanisms that could result in significant graft injury and failure: (1) activation of complement cascade can cause direct injury to the capillary endothelium, (2) antibody-dependent cell-mediated cytotoxicity by natural killer cells, (3) opsonization and increased antigen presentation, and (4) activation and proliferation of endothelial cell. FcR, Fc receptor; i-KIR, inhibitory KIR; a-KIR, activating KIR; APC, antigen-presenting cell; C1q, a complement complex.

The Y-shaped structure of IgG antibodies provides a bifunctional capacity to initiate and regulate host defense mechanisms in the following ways: antigen binding through the Fab (antigen-binding fragment) portion of the antibody and the interaction with immune cells and complement proteins (fragment crystallizable or Fc). Following DSA binding to the allograft endothelium, at least four distinct cellular and humoral mechanisms exert significant graft injury and failure (Figure 1).

### Activation of the Complement Cascade

Complement fixation by antibody is essential for the pathogenesis of acute and hyperacute rejection (13). The binding of DSA to a cell surface antigen expressed on the allograft may trigger the classical complement pathway, a component of the innate immune system (14). The complement component C1q binds to structures in two or more Fc domains of IgM or IgG, which causes C1q to undergo a conformational change, which allows the enzymatic components C1r and C1s in the collagenous portion of the antibody-bound C1q to cleave C4 molecules (13). This initiation step then leads to the recruitment of other proteins in order to form the C3 convertase protein complex. Activation of C3 leads to the generation of two pro-inflammatory anaphylatoxins, C3a and C5a, and the membrane attack complex that eventually forms a pore in the membrane of the target and induces cell death. The classical pathway is only one of three methods of complement activation, the others being the alternative pathway and the lectin pathway, and all three pathways converge at the point of C3 cleavage (13). C4d is a split product of C4 groups, which remains covalently bound to the tissue and is thereby a durable *in situ* marker of complement activation. Detection of C4d deposition in capillaries has proved to be the most reliable marker of ABMR (15). Although the peritubular capillary C4d detection is important, it is not necessary to diagnosis ABMR, since the presence of DSA has the potential to cause transplant glomerulopathy and graft loss due to complement-independent mechanisms (16).

### Antibody-Dependent Cell-Mediated Cytotoxicity

In addition to activating complement-dependent cytotoxicity against the allograft, antibodies can mount immune responses through interacting with Fc receptors (FcγRs), which are widely expressed throughout the hematopoietic system (17). Three different classes of FcγRs, known as FcγRI (CD64), FcγRII (CD32) with A, B, and C isoforms, and FcγRIII (CD16) with A and B isoforms, have been recognized in humans. Except FcγRIIIB that is present mainly on neutrophils, all other FcγRs are activating receptors. Innate immune effector cells, including monocytes, macrophages, dendritic cells (DCs), basophils, and mast cells, coexpress activating and inhibitory FcγRs, whereas B-cells express the inhibitory receptor FcγRIIB (17). Natural killer (NK) cells, particularly those with CD56^dim^ CD16^+^ phenotype express activating low-affinity FcγRIIIA. NK cells are regarded as the key effector cells mediating antibody-dependent cell-mediated cytotoxicity (ADCC) function since NK cells are the only subset that do not coexpress the inhibitory FcγRIIB (18).

Infiltration of recipient NK cells into the renal (19), cardiac (20), lung (21), and liver (22) allografts shortly following transplantation have been observed indicating a role for human NK cells in solid organ transplantation. Direct evidence for the role of NK cells in microcirculation injury during ABMR comes from the findings of NK cells and NK cell transcripts in kidney biopsies from patients with donor-specific HLA antibodies (23, 24). Mechanistic studies confirming the role of DSA-dependent NK cell-mediated cytotoxicity in organ allograft rejections is lacking (25). However, clinical trials with cancer therapeutic antibodies have shown that the induction of NK cell-mediated ADCC have direct bearing on organ allograft rejection. For example, rituximab, a chimeric mouse-human IgG1 monoclonal antibody that recognizes the CD20 antigen expressed on mature B-cells, is used to treat patients with B-cell lymphomas and autoimmune disorders. Both quantitative and qualitative differences in NK cell function are correlated with rituximab clinical activity, suggesting that ADCC performed by NK cells may be a primary mechanism of rituximab activity (26). Furthermore, responses to rituximab may depend on polymorphisms present in the FcRIIIA receptor, a receptor mainly expressed on NK cells (27, 28). Several other antibodies are currently being evaluated in the clinic and, for many of them, their effect seems to be mediated at least in part by NK cell-mediated ADCC (29). In addition to ADCC, on FcγRIIIA stimulation, NK cells produce cytokines and chemokines, including interferon-γ (IFN-γ), which may induce HLA expression on endothelial cells, thus providing more antigenic targets for antibodies and shortening graft survival (30). More understanding of FcγRIIIA-mediated regulation of NK cell function is critical in order to define the role of NK cell transcripts in kidney biopsies from patients with donor-specific HLA antibodies.

### Opsonization and Promotion of Antigen Presentation

In addition to their well-defined roles in triggering ADCC by NK cells, FcγRs regulate antigen presentation, immune complex-mediated maturation of DCs, B cell activation, and plasma cell survival, and therefore, FcγRs ultimately regulate the production and specificity of their ligands, antibodies (31). The ligation of Fab of the DSA to the alloantigen attracts phagocytes (neutrophils, monocytes, macrophages, and DCs) to infiltrate into the allograft. The Fc fragment of the antibody binds to an Fc receptor on the phagocyte, facilitating receptor-mediated phagocytosis, which accelerates the kinetics of the phagocytosis process (32). Phagocytosis initiates specific mechanisms that result in trafficking of the antigen–IgG immune complexes into compartments from which the antigens are processed into peptides for HLA class I and class II presentation to CD8^+^ and CD4^+^ T cells, respectively, thereby FcγRs bridge the humoral and cellular branches of the adaptive immune response.

### Activation of Endothelial Cells

The *in vitro* experiments of anti-HLA antibody ligation have shown that HLA class I molecules expressed by endothelial cells stimulates endothelial cell activation and proliferation (33, 34). Endothelial cell proliferation may be at least partly causative of arterial intimal thickening that is characteristics of chronic allograft rejection.

## Natural Killer Cells Link Innate and Adaptive Immunity

Natural killer cells are the third population of lymphocytes defined by the CD3^−^ CD56^+^ cell surface phenotype, and they represent 5–25% of the mononuclear cell fraction of normal human peripheral blood (35). NK cells share several features with CD8^+^ cytolytic T-lymphocytes in their development, morphology, cell surface phenotypes, killing mechanism, and cytokine production (36). NK cells were originally described as innate lymphocytes capable of lysing target cells quickly by direct cytotoxicity in an antigen-independent manner without the “priming” period required by T-cells (37). NK cells are recognized to express a sophisticated repertoire of activating and inhibitory receptors that are calibrated to ensure self-tolerance, while exerting early assaults against virus infection (38) and tumor transformation (39). In addition to cytolytic functions, NK cells produce high levels of IFN-γ and a wide range of pro-inflammatory cytokines and chemokines, which contribute to the shaping of adaptive immune responses (40). Recently, NK cells have been shown to mount antigen-specific immunologic memory, a hallmark characteristic of adaptive immunity (41). Having properties of both innate and adaptive immunity, NK cells spontaneously lyse target cells, as well as function as regulatory cells influencing subsequent antigen-specific T-cell and B-cell responses.

## NK Cells in Solid Organ Transplantation

Experiments with rodent models clearly indicate a role for NK cells in acute and chronic allograft rejection (42–44). The most convincing evidence of NK cell-mediated rejection was observed with the heart allograft missing-self-MHC class I in CD28-deficient recipient mice; in this model, rejection is prevented by depletion of host NK cells (45). NK cells play a crucial role in mediating long-term kidney allograft injury (46). Currently, used clinical regimen of immunosuppressive agents such as cyclosporine A (47), FK506 (48), mycophenolate mofetil (49), azathioprine (50), and rapamycin (51) appears not to abrogate NK cell function. NK cell number and the cytotoxicity function were preserved to a greater extent in a regimen of tacrolimus and mycophenolate mofetil than they were with cyclosporine A and azathioprine 12 months after kidney transplantation (52). Even in the presence of polyclonal anti-thymoglobuline antibody that depleted T and NK cells transiently, the NK cell effector function is preserved after kidney transplantation (53).

## NK Cells Use a Complex Receptor–Ligand System to Distinguish Non-Self from the Self

Natural killer cells use very complex and specific receptor–ligand system that integrates signals triggered by an array of inhibitory and activating receptors, which trigger cytotoxicity and the secretion of chemokines and cytokines (54, 55). Unlike T- and B-lymphocytes, NK cells do not express receptors that require somatic gene rearrangements to generate receptor diversity and specificity. Instead, NK cells express a wide array of conventional germline-encoded receptor families with inhibitory or activating functions that scan for missing-self, induced-self, and altered-self on target cells. The well-characterized NK cell receptor gene families include killer cell immunoglobulin-like receptors (KIR), killer cell lectin-like receptors, leukocyte immunoglobulin-like receptors, and natural cytotoxicity receptors (56–59).

## Killer Cell Immunoglobulin-Like Receptors and HLA Class I Ligands

**Figure 2:**
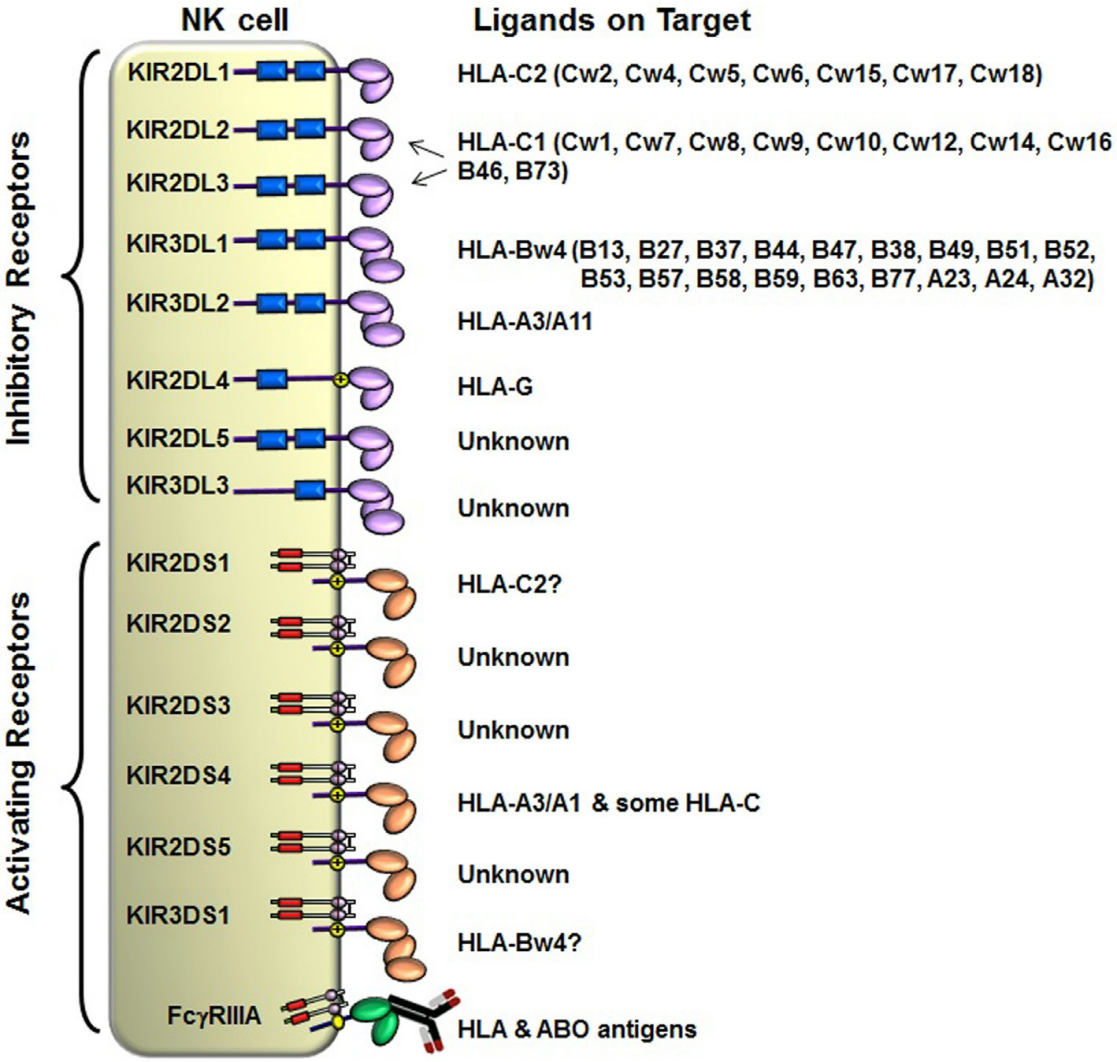
**Killer cell immunoglobulin-like receptor (KIR) and human leukocyte antigen (HLA) class I ligands**. Fourteen distinct KIRs have been characterized in humans that comprise either 2 or 3 (2D or 3D) Ig-like domains and either a long (L) or short (S) cytoplasmic tail. Six KIRs are activating types and the remaining KIRs are inhibitory types. The cytoplasmic tails of the inhibitory KIRs carry an ITIM motif (shown as blue boxes) that trigger inhibitory signals upon binding to distinct HLA class I ligands. The short-tailed activating KIRs lack ITIM, but carry a positively charged amino acid residue in the transmembrane region (shown by yellow circle with + mark) that allows the interaction with an adopter chain DAP-12. The DAP12 contains ITAM motifs (shown as red boxes), which trigger activating signals upon the short-tailed KIR bound to a relevant ligand.

**Figure 3:**
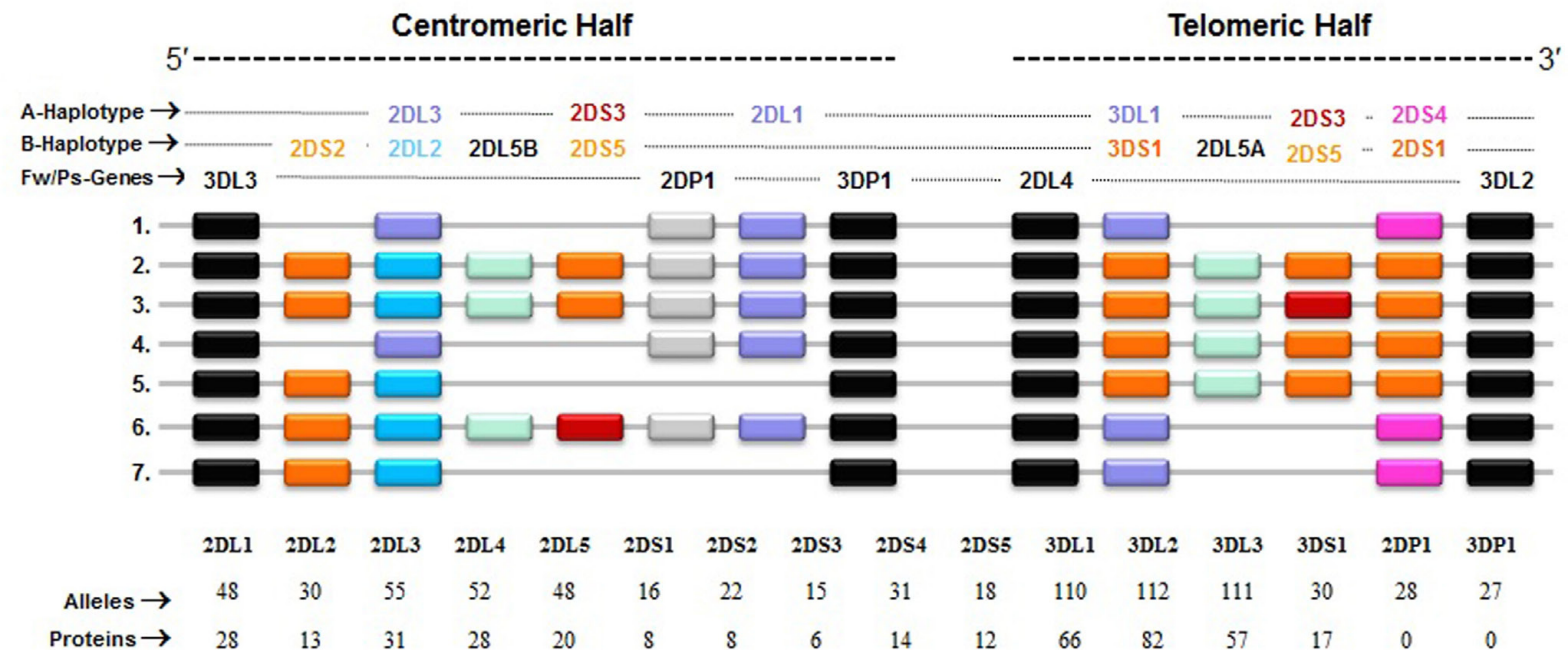
**Killer cell immunoglobulin-like receptor (KIR) haplotypes vary in gene content**. Map of common *KIR* haplotypes in Caucasian populations. Each box represents a *KIR* gene. Haplotype#1 represents group-A *KIR* haplotype and the remainder are group-B haplotypes. The framework (Fw) genes, present in all haplotypes are shown in gray; genes encoding activating KIR are in pink (A haplotype-specific) or red/orange (B haplotype-specific); those for inhibitory receptors are in purple (*A* haplotype-specific) or blue (B haplotype-specific); and Pseudogene (Ps) *2DP1* is in white. All *KIR* genes are polymorphic, and the number of alleles and proteins characterized for each *KIR* gene is indicated.

The KIRs are crucial for human NK cell development and function (56, 58, 60) (Figure 2). The *KIR* gene family does not exist in rodents and found only in primates, and therefore *KIR* genes are considered to be originated recently and evolved rapidly (61, 62). The *KIR* gene family consists of 16 highly homologous genes clustered at the leukocyte receptor complex on chromosome 19 (63, 64) (Figure 3). Seven of them encode inhibitory KIRs (3DL1–3, 2DL1–3, and 2DL5), six encode activating KIRs (3DS1, 2DS1–2DS5), one encode a KIR that can trigger both inhibitory and activating signals (2DL4), and two are pseudogenes (*2DP1* and *3DP1*) that do not encode a cell surface receptor. By recognizing specific HLA class I ligands, the inhibitory KIRs trigger signals that stop NK cell function, while the ligands for activating KIRs are not elucidated. Genetic association studies suggest the possibility of activating KIRs recognizing cell surface determinants expressed following infection or tumor transformation, or under certain physiological, stress such as transplantation (65).

In general, humans have two copies of each autosomal gene, one per chromosome. However, due to deletion and duplication, the basic diploid rule does not apply to *KIR* gene family. The number and type of *KIR* genes vary substantially between haplotypes, and all *KIR* genes display sequence polymorphism (66) (Figure 3). On the basis of gene content, *KIR* haplotypes are broadly classified into two groups (67). Group A haplotypes have a fixed gene content (*KIR3DL3–2DL3–2DP1–2DL1–3DP1–2DL4–3DL1–2DS4–3DL2*) that encode four inhibitory KIRs, 2DL1, 2DL3, 3DL1, and 3DL2, specific for four major HLA class I ligands, C2, C1, Bw4, and A3/A11, respectively, and an activating KIR 2DS4, which is weakly specific for some HLA-C allotypes (C1 or C2 epitope), as well as the HLA-A3/11 epitope (Figure 2). In contrast, group B haplotypes are variable both in numbers and combinations of *KIR* genes, and comprising several genes (*2DL2, 2DL5, 2DS1, 2DS2, 2DS3, 2DS5*, and *3DS1*) that are not part of the A haplotype (63, 68, 69). Moreover, B haplotypes possess KIRs that have no binding to HLA class I ligands, such as KIR2DL5, 2DS2, 2DS3, and 2DS5. While group A haplotypes contain only *KIR2DS4* as an activating gene, group B haplotypes contain up to five activating *KIR*s – *KIR2DS1, 2DS2, 2DS3, 2DS5*, and *3DS1*. Inheritance of paternal and maternal haplotypes comprising different *KIR* gene contents generates human diversity in *KIR* genotypes (70). For example, homozygotes for group A haplotypes have only seven functional *KIR* genes, while the heterozygotes for group A and certain group B haplotypes may have all 14 functional *KIR* genes. All human populations have both group A and B *KIR* haplotypes, but their incidences vary substantially among populations (71–74). The A and B haplotypes are equally distributed in Africans and Caucasians, while the A haplotype is overrepresented in Northeast Asians (Chinese, Japanese, and Koreans) and the B haplotype occurred most frequently in the indigenous populations of India, Australia, and America (75).

## NK Cells Use Three Distinct Mechanisms to Injure Allograft Tissue

**Figure 4:**
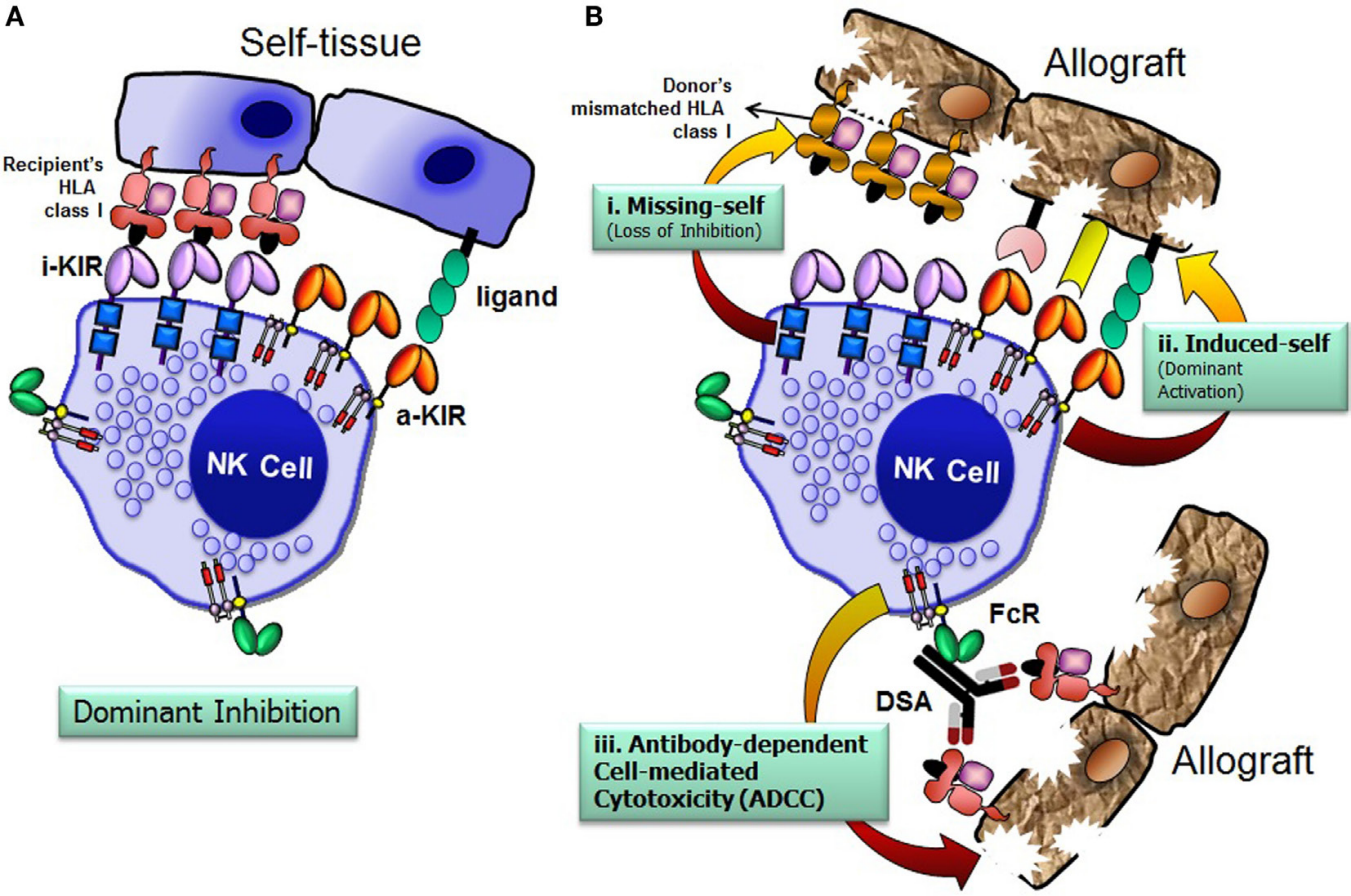
**Natural killer (NK) cells distinguish allograft lacking self-human leukocyte antigen (HLA) class I molecules**. The net signal integrated from the inhibitory and activating receptors determines the effector function of NK cells. NK cells spare healthy cells that express high levels of HLA class I molecules and low levels of ligands for activating receptors **(A)**. Recognition of cognate HLA class I ligands on a healthy cell by inhibitory receptors expressed by NK cells prevents lysis of the healthy cell **(A)**. NK cells recognize and injure allograft that has either disparate HLA class I (i. missing-self; loss of inhibition), express high levels of ligands for activating receptors (ii. induced-self; dominant activation), and/or iii. donor-specific HLA antibody-dependent cell-mediated cytotoxicity **(B)**. FcR, Fc receptor; i-KIR, inhibitory KIR; a-KIR, activating KIR; DSAs, donor-specific antibodies.

The recipient NK cells can recognize and respond against the allograft by three possible mechanisms: missing-self recognition, induced-self recognition, and ADCC (Figure 4) (76). Because NK cells circulate in a state that can spontaneously deliver effector function, it is critical that they do not attack surrounding healthy cells. To prevent such detrimental autoreactivity, NK cells express an array of inhibitory receptors recognizing self-HLA class I molecules (Figure 4A). Expression of four distinct HLA class I molecules (HLA-A, -B, -C, and -E) on normal healthy cells provides ligands for various inhibitory receptors of NK cells and, consequently, are resistant to NK cell attack. Downregulation of HLA class I expression due to certain viral infections, neoplastic transformations, or absence of relevant HLA class I ligands on the allograft at the setting of allogeneic transplantation, alleviates inhibitory signals, permitting NK cells to eliminate these unhealthy or allogeneic target cells, a phenomenon originally described as the “missing-self” hypothesis (77) (Figure 4B, i). In addition to the “missing-self” mechanism, the expression of ligands for activating receptors on stressed target cell surface might also contribute to NK cell attack, known as “induced-self” recognition (Figure 4B, ii). The activation receptors can directly recognize stress-induced ligands associated with certain physiological conditions, such as infection, tumor transformation, and transplanted allograft (58, 78). The third mechanism is mediated *via* an ADCC (Figure 4B, iii), in which NK cells are activated through the low-affinity Fc receptor for IgG FcγRIIIA (CD16) by binding to the Fc portion of DSA. In summary, the NK cells discriminate the stressed unhealthy cells or allograft from the healthy self by gauging the net input of activating and inhibitory signals perceived from the NK cell receptors upon their interactions with target cell ligands.

## Clonal Expression of KIR and Acquisition of NK Cell Tolerance and Responsiveness

Similar to T- and B-lymphocytes, NK cells are developed from CD34^+^ hematopoietic stem cells in the bone marrow and undergo terminal maturation in secondary lymphoid tissues (79–81). A signature feature of KIR is their clonal expression on NK cells, so that each NK cell clone in a person expresses only a portion of the genes within their *KIR* genotype (82–84). Stochastic expression of different combinations of receptors by NK cells results in this repertoire of NK clones with various ligand specificities. Once a given KIR is expressed on an NK cell clone, it is maintained in a stable way in the progeny of the clone. The process that establishes these clonal patterns is based on epigenetic regulation by DNA methylation and histone modifications (85–87).

Because *KIR* and *HLA* genes are located on different chromosomes (*KIR* on chromosome 19 and *HLA* on chromosome 6), *KIR* genes are inherited independently from *HLA* genes, and KIR may be expressed in the absence of their HLA ligands (88). Most, but not all, NK cell clones in peripheral blood express at least one inhibitory receptor for self-HLA class I (82). Only those NK cell clones expressing at least one inhibitory KIR specific for self-HLA class I molecule are “licensed,” or functionally active, to eliminate target cells that have downregulated or which are missing the respective HLA class I ligands (74, 78, 89–91). A conceivable explanation for NK cell licensing is that inhibitory KIRs, upon specific interaction with self-HLA class I allotypes, deliver a signal resulting in NK cell maturation and acquisition of effector function. NK cells lacking inhibitory receptors for self-HLA class I molecules are considered to be developmentally immature, “unlicensed,” and substantially hyporesponsive to HLA class I-negative targets (74, 89, 92, 93). Therefore, the NK cell responsiveness is most fundamentally distinguished by the presence or lack of inhibitory KIR for self-HLA class I. Licensed NK cells further vary in effector function quantitatively according to the strength of the inhibitory KIR and HLA interactions and the copy number of the corresponding inhibitory *KIR* and *HLA* genes (94–96). In summary, KIR receptor–HLA class I ligand interactions at the developmental stage set the functional threshold for NK cell and regulate NK cell effector function.

## KIR–HLA Interactions Can Modulate the DSA-Dependent NK Cell-Mediated Cytotoxicity Against Organ Allograft

Polymorphic variation among the *KIR* and *HLA* class I genes and their resulting impact on the KIR and HLA interaction constitute a major source of variability in NK cell responsiveness (94–96). These differences influence clinical outcomes in diverse settings, including monoclonal antibody therapy for lymphoma (97), transplantation for hematological malignancies (98), kidney transplantation (99, 100), and other settings in which NK cell involvement contributes to disease control and clinical responses (101–105). However, not all KIR^+^ licensed NK cells are equivalent, as polymorphic diversity in the *KIR* and *HLA* genes underlie significant variation in binding strength and specificity, which quantitatively influence licensing, inhabitability, and ADCC (104, 106–111).

However, studies supporting a role for licensing in human ADCC are limited. A prominent role for KIR3DL1/HLA-Bw4 interactions in licensing NK cells for CD16-mediated effector function was published recently (112). When individuals expressed both inhibitory KIRs that interact with HLA-C and the corresponding HLA-C ligand, their NK cells exhibited greater general and Fc receptor-mediated effector functions than NK cells from those individuals lacking the relevant HLA-C ligand (74). Similarly, expression of KIR3DL1, an inhibitory KIR that interacts with the HLA-Bw4 public epitope, was associated with higher NK cell cytotoxicity and IFN-γ production upon exposure to HLA class I-deficient target cells when the NK cells were isolated from HLA-Bw4 donors (94, 112). Therefore, the interindividual differences in compound *KIR* and *HLA* class I ligand genotypes associated with differences in NK cell reactivity would impact DSA-mediated NK cell ADCC against the organ allograft. The individualized assessment of the recipient’s KIR, FcR, HLA types, HLA antibodies, and the donor’s HLA types at the molecular and functional levels have the potential to distinguish between mechanisms that could guide identification of new therapeutic targets for ABMR.

## Concluding Remarks

The complement-independent mechanisms that lead to the ABMR of kidney allografts remain poorly understood. Recent studies finding a link between ABMR and abundance of NK cell molecular signatures in transplant biopsy suggest relevance of NK cells as innate immune cytotoxic effectors to antibodies through ADCC. However, the direct pathogenic role of donor-specific HLA antibody-mediated NK cytotoxicy in transplant rejection remains poorly documented by mechanistic studies. NK cells use very complex and specific germline-encoded KIR receptor and HLA class I ligand system that integrate signals triggered by an array of inhibitory and activating receptors, which set NK cell maturation and acquisition of effector function. Moreover, the FcγRIIIA polymorphisms and expression levels can also modulate NK cell activation against allograft. Future studies that integrate both recipient factors (such as KIR receptors, HLA class I ligands, FcγRIIIA polymorphisms, and donor-specific HLA antibodies) and donor factors (such as HLA class I ligand compatibility with recipient) that establish variable KIR–HLA conditioned NK cell-FcγRIIIA-antibody–antigen interactions will identify potential interindividual variability of humoral alloimmune responses.

## Author Contributions

The author confirms being the sole contributor of this work and approved it for publication.

## Conflict of Interest Statement

The author declares that the research was conducted in the absence of any commercial or financial relationships that could be construed as a potential conflict of interest.
